# Biomarkers for screening of lung cancer and pre-neoplastic lesions in a high risk Chilean population

**DOI:** 10.1186/0717-6287-47-62

**Published:** 2014-11-25

**Authors:** Marta I Adonis, Jose Díaz, Veronica R Miranda, Marco Chahuan, Alcides Zambrano, Hugo C Benitez, Monica Campos, Pablo Avaria, Ulises Urzúa, Pedro Marín, Mariela Gohurdett, Yasna Cisterna, Lionel Gil

**Affiliations:** Faculty of Medicine, University of Chile, Avenida Independencia 1027, Santiago, Chile; Barros Lucos Trudeau Hospital, Santiago, Chile; San Borja Arriaran Hospital, Santiago, Chile; Antofagasta Regional Hospital, Antofagasta, Chile; La Portada Clinic, Antofagasta, Chile

**Keywords:** Early detection, Biomarkers, Lung cancer

## Abstract

**Background:**

The mortality of lung cancer (LC), increases each year in the world, in spite of any advances, in development of new drugs to advance stages of LC. The high incidence of LC has been associated with smoking habit, genetic diversity and environmental pollution. Antofagasta region has been reported to have the highest LC mortality rate in Chile and its inhabitants were exposed to arsenic in their drinking water in concentrations as high as 870 μg/L. Non-invasive techniques such as biomarkers (Automatic Quantitative Cytometry: AQC and DR70) and Auto Fluorescence Bronchoscopy (AFB) might be potentially useful as a supplementary diagnostic approach and early detection. Early detection is one of the most important factors to intervene and prevent cancer progression in LC. This is a work of an ongoing prospective bimodality cancer surveillance study in high risk LC volunteers. Enrolment was done in subjects from Antofagasta and Metropolitan regions. In addition, we enrolled subjects who were suspected of having lung cancer. AQC, DR70 and AFB were used as tools in the detection of pre-neoplastic (PNL) and neoplastic lesions (NL).

**Results:**

Half of the samples, classified as suspicious by AFB, were confirmed as metaplasia or dysplasia by histopathology. For LC, DR70 showed a higher sensitivity (95.8%) and specificity (91.9%) than AQC. However, for PNL AQC showed a higher sensitivity (91.9%) than DR70 (27.3%), although both with low PPV values. As a pre screener, both biomarkers might be employed as complementary tools to detect LC, especially as serially combined tests, with a sensitivity of 60% and a PPV of 65.2%. Additionally, the use of parallel combined tests might support the detection of PNL (sensitivity 91.2%; PPV 49.1%).

**Conclusion:**

This work adds information on cellular and molecular biomarkers to complement imaging techniques for early detection of LC in Latin America that might contribute to formulate policies concerning screening of LC. Supported by INNOVA-CORFO, Chile.

## Background

The high incidence of lung cancer (LC) has been associated with cigarette smoking, however genetic diversity and environmental pollution must also be considered as risk factors, especially in those cities highly exposed to environmental carcinogens. During 2008, 1.52 million new LC cases and 1.31 million deaths were reported worldwide [1]. Early detection is one of the most important factors to prevent cancer progression in lung cancer (LC). In this context, non-invasive techniques such as collection of induced sputum samples for conventional or automatic quantitative cytometry (AQC) and detection of serum tumour markers might be potentially useful as a supplementary diagnostic approach.

There were more than 2,500 LC cases and 1,900 LC related deaths during 2007 in Chile. Between 1990 and 2008, the national mortality rate/100,000 habitants increased from 10.8 to 14.6 for both genders. For the period of 2003–2007, the Antofagasta region (Northern Chile) showed a mortality rate of 30.8/100,000, the second highest mortality rate after skin cancer [2–6]. The mortality rate, might be related with environmental factors, like to air pollution and or Arsenic exposition. Many carcinogenic compounds present in cigarette smoke, have also been identified in airborne particles in different cities around the world [7–15] including Santiago. Has been described that Benzo(a)pyrene (BaP), one of PAHs associated to cigarette smoke, induce lung cancer through DNA damage [16–18]. High levels of BaP and individual host susceptibility might determine a high levels of diolepoxide (BPDE)-DNA adduct. It has been postulated that natural compounds like arsenic (As), enhance the BPDE-DNA adduct induced mutagenesis, suggesting that As might act as a co-mutagen to promote the development of human LC [19–21].

Santiago’s inhabitants have been exposed to high levels of BaP, reaching the 4.9 ng/m^3^, during the year 1996 [6]. On the other hand, Antofagasta region had drinking water arsenic (dw-As) concentration of 90 μg/L before 1958 [22]. During 1958–1970, dw-As concentration reached 870 μg/L. Since then, As levels have progressively decreased to the new Chilean standard (10 μg/L). The antecedents of dw-As in the Antofagasta region shows that during 1956 the region received drinking water from the Siloli river, with As concentration of 90 μg/L. Later, the increasing mining activity led to rapid growing of the population and a new adduction of the Toconce river, with dw-As concentration of 823 μg/L, causing an increasing dw-As concentration in the region, exposing and affecting to more than 300.000 habitants [23].

The implementation of early-detection technologies and prognostic biomarkers are imperative in Chile, specifically in cities that are highly exposed to environmental carcinogens, like to PAHs and or Arsenic [22, 24]. This work has been done in Antofagasta and Metropolitan regions in order to evaluate two biomarkers AQC and DR70 (Onko Sure) as tools in detection of LC and detection of pre-neoplastic lesions related with LC. Previously, the researchers of this work used the biomarker DR70, as screening tool of LC in advance stages but not in pre neoplastic lesions, in a population exposed historically to As in drinking water, showing that this biomarker might provide relevant information to identify individuals with a lung cancer [19, 20]. Additionally, the researchers have been working with sputum specimen in order to study genomic alterations in a Chilean population with high risk of LC (manuscript in preparation). Then, AQC has been considered as complementary tool in order to have additionally information associated to malignancy of LC in the same kind of sample (sputum specimen).

AQC, is a quantitative morphometric analysis of the amount and distribution of DNA in sputum cells using normal epithelial cells [25]. Onko-Sure™, is an in vitro diagnostic test that has been described to effectively monitor and/or detect solid cancerous tumours [26, 27]. Auto fluorescence Bronchoscopy (AFB), is a technique that exploits differences in fluorescence properties of normal and abnormal bronchial mucosal tissues for the detection of preinvasive and micro-invasive bronchial lesions, which might otherwise be invisible on White Light Bronchoscopy (WLB) [28–32].

## Results

In Santiago 56.7% were current smokers (N = 127), 35.3% were ex-smokers (N = 79) and 8% (N = 18) were never smokers. For Antofagasta, 37.1% (N = 52) were current smokers, 36.4% (N = 51) ex-smokers and 26.4% (N = 37) were never smokers. According to the smoking index (packages per year, p/y) [33], most of 43% the volunteers included in this study were classified as medium (> ½ -20 p/y) or intense (>20 -40 p/y) smokers from among either current or ex-smokers.

### AQC of induced sputum and DR70

Table 1 shows the likelihood of malignancy by AQC and DR70. For AQC, 27.2% of the subjects showed an increased likelihood of malignancy and 26.6% an undetermined likelihood of malignancy (a score of 3.9 < 4.6). Among samples with increased AQC, 15.4% and 11.8% were negative and positive for DR70 (>1 μg/mL), respectively. Samples with undetermined AQC showed 22.5% and 4.1% of negative and positive DR70 levels; respectively. Additionally, among 46.2% of AQC samples that showed decreased likelihood of malignancy, 42.3% were negative and 3.9% were positive for DR70.

**Table 1:** **AQC and DR70 correlation and percentage (N) for each LC risk interval**

Likehood of malignance	AQC % of sample	Likehood of malignance DR70 % of sample (N)	
		Increased	Decreased
**Increased**	27.2 (99)	11.8 (43)	15.4 (56)
**Undetermined**	26.6 (97)	4.1 (15)	22.5 (82)
**Low**	46.2 (168)	3.9 (14)	42.3 (154)

Likehood of malignance.

AQC: Increased ≥ 4.6; Undetermined ≥ 3.9 to < 4.6; Low ≤ 3.8.

DR70: Increased ≥ 1.0; Decreased < 1.0.

Table 2 shows results related with the bronchoscopy procedure, applied to 98 volunteers. The WLB (Table 2A) showed a 23.47%, 61.22%, 3.06% and 12.25% with normal condition, inflammation, suspicious condition and LC; respectively. According to WLB, suspicious cases are related with cancer in situ (CIS), however the Histopathology Assay (HA) classified all of these cases as inflammation. The HA results showed that a high percentage of the normal cases according to WLB results, were hyperplasia (56.52%) and metaplasia (21.74%) and only 17.39% of them were truly normal. For the sites that were recognized to be inflammation on WLB, the HA confirmed 28.33% of them as inflammation and the rest were distributed as normal (11.67%), hyperplasia (48.33%) and metaplasia (6.67%).

**Table 2:** **Relationship between White Light Bronchoscopy (WLB) (A), Autofluorescence Broncoscopy (AFB) (B) and Histopathology Assay (HA)**

**A**	**HA % (N)**						
**Variable % (N)**	**WLB % (N)**	**Normal**	**Inflammation**	**Hyperplasia**	**Metaplasia**	**Dysplasia**	**LC**
**Normal**	23.47 (23)	17.39 (4)	4.35 (1)	56.52 (13)	21.74 (5)	0	0
**Inflammation** (a)	61.22 (60)	11.67 (7)	28.33 (17)	48.33 (29)	6.67 (4)	5.0 (3)	0
**Suspicious** (b)	3.06 (3)	0	100 (3)	0	0		0
**LC**	12.25 (12)	0	0	0	0	25.0 (3)	75.0 (9)
**TOTAL**	**(98)**	**(11)**	**(21)**	**(42)**	**(9)**	**(6)**	**(9)**
**B**	**HA% (N)**						
**Variable % (N)**	**AFB % (N)**	**Normal**	**Inflammation**	**Hyperplasia**	**Metaplasia**	**Dysplasia**	**LC**
**Normal**	18.36 (18)	22.22 (4)	5.56 (1)	61.11 (11)	11.11 (2)	0	0
**Inflammation** (a)	57.14 (56)	12.5 (7)	30.35 (17)	50.0 (28)	5.36 (3)	1.79 (1)	0
**Suspicious** (b)	12.25 (12)	0	25.0 (3)	25.0 (3)	33.33 (4)	16.67 (2)	0
**LC**	12.25 (12)	0	0	0	0	25.0 (3)	75.0 (9)
**TOTAL**	98	**11**	**21**	**42**	**9**	**6**	**9**

(a): Abnormal or Inflammation (Erythema, swelling or thickening of bronchial mucosa, airway inflammation, and Fibrosis) [31].

(b): Suspicious for pre invasive or preneoplastic lesion for AFB and cancer in situ (CIS) for WLB.

On other hand, AFB classified 18.36%, 57.14% and 12.24%of the cases as normal, inflammation and suspicious for LC; respectively (Table 2B). The HA showed that within of the 18.36% that were classified by AFB as normal, 22.22% was really normal and the rest were distributed as inflammation (5.56%), hyperplasia (61.11%) and metaplasia (11.11%). The inflammations that were diagnosed according to AFB (57.14%) were confirmed by HA in 30.35% and the rest were classified as normal, hyperplasia, metaplasia and dysplasia (12.5%, 50.0%, 5.36% and 1.79%; respectively).

For the suspicious biopsies (invasive or pre-neoplastic lesions), according to AFB (12.2%), HA confirmed 33.33% of them as metaplasia and 16.67% as dysplasia. The rest were classified by HA as inflammation (25.0%) and hyperplasia (25.0%). Additionally, for the patients that were suspicious for LC, with the actual diagnosis in the study and without any treatment or previous additional assays, AFB results confirmed the WLB diagnosis in 100% [12]. However, only 75% of them (9 cases out of 12) were confirmed by the HA as LC, a 25% were classified as dysplasia. Additional studies, such as CT, confirmed all 12 cases as LC. Therefore, of a total of 98 volunteers with AFB, 42 cases were related with hyperplasia (42.9%), nine cases with metaplasia (9.2%), three cases with dysplasia (3.1%) and 12 cases as LC (12.2%). AFB identified the PNL (metaplasia and dysplasia) better than WLB.

**Figure 1:**
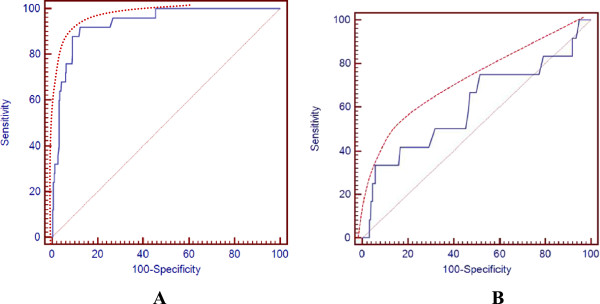
**DR70 ROC curve for LC (A) and Pre-neoplastic Lesions related with LC (B).**

Figure 1 shows the overall ROC performance for DR70 in LC (Figure 1A,) and preneoplastic lesions (PNL) (Figure 1B). The empirical specificity and sensitivity of this test for LC were 91.87% (95% confidence interval [CI]: 88.1–94.8%) and 95.83% (95% CI: 78.9–99.9%); respectively. The Predictive Positive Value (PPV) was 50.0 (95% CI: 35.1–65.1%) with a Predictive Negative Value (PNV) of 99.6% (95% CI: 97.8-100%). The LR + (Positive Likelihood Ratio) was 11.79 (95% CI: 10.8-12.9%), increasing further with higher thresholds. According to these results, a patient’s positive test result was considered as someone with 11.8 times higher risk of the presence of LC, as compared to a normal individual. In contrast, the LR- for was 0.045 or 1/22 (95% CI: 0.006–0.3%), meaning that a negative test result corresponded with a decrease in the subject’s odds of disease by a factor of 22. In conclusion, the ROC curve of DR70 for LC was slightly skewed toward higher sensitivity, although both sensitivity and specificity were quite high. However the LR + and LR- results suggested that the test identified subjects with higher risk of lung cancer but did better for ruling out LC. The biomarker DR70 showed a higher sensitivity and specificity than the marker CYFRA21-1, with a relative high diagnostic value for lung carcinoma. CYFRA21-1 has showed a sensitivity of 56.3% and a specificity of 86.7%, in serum of LC patients with malignant pleural effusion [34]. Additionally, a recently study of CYFRAA21-1, in serum of LC patients, showed a sensitivity and specificity of 46.21% and 97.14%, respectively; showing a higher sensitivity for Squamous lung cancer (71.43%) than Adenocarcinoma (29.41%) and Small Cell Lung Cancer (12.5%) [35].

For PNL, the test showed a specificity and sensitivity of 91.87% (95% CI: 88.1–94.3%) and 27.27% (95% CI: 6.0–61.0%); respectively. The Predictive Positive Value for PNL was 11.5% (95% CI: 2.4–30.2%) and the Predictive Negative Value was 97.0% (95% CI: 94.2-98.7%). The LR was 3.36 (95% CI: 1.3-8.8%) and the LR- was 0.79 or 1/1.27 (95% CI: 0.5–1.3%). According to sensitivity, specificity, PPV, PNV, LR + and LR-, DR70 by itself might identify patients with PNL with a sensitivity of 27.27%. However, the test might be better in order to identify subjects with a low likelihood of PNL, with a specificity of 91.87%.

**Figure 2:**
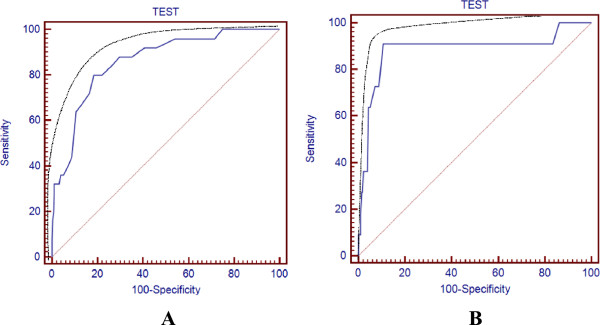
**AQC ROC curve for LC (A) and Pre-neoplastic Lesions related with LC (B).**

The Figure 2 shows, the overall ROC performance for AQC for LC (Figure 2A) and Preneoplastic lesions (PNL) (Figure 2B). AQC for LC (Figure 2A), showed a sensitivity and specificity of 64% (95% confidence interval [CI]: 42.5%–82%) and 89.4% (95% confidence interval [CI]: 85.2%–92.7%), allowing detecting and confirming positive and negative cases with high precision. The PPV, was 34.8% (95% CI: 21.2%–50.4%) with a PNV of 96.6% (95% CI: 93.6 -98.6). The LR + was 6.04 (95% CI: 4.5-8.1), increasing further with higher thresholds. According to these results, a patient’s positive test result would be someone with 6.04 time higher risk of presence of disease. In contrast, the LR- was 0.40 or 1/2.5 (95% CI: 0.2– 0.8), meaning that a negative test result corresponded with decrease in the subject’s odds of disease by around a factor of 2.5.

Kemp et al. [25], published for LC, an sensitivity of 40% (95% CI: 35%–46%) and empirical specificity of 91% (95% CI: 89%–93%), with a threshold of 5.0. Our results, with thresholds of 5.0, showed a sensitivity of 36% (95% CI: 18%–57.5%) and specificity of 95.76% (95% CI: 92.7%–97.8%).

For PNL, the Figure 2B shows a sensitivity of 90.91% (95% CI: 58.7%–99.8%) and an empirical specificity of 89.40% (95% CI: 85.2%–92.7%). The PPV for PNL, was 25% (95% CI: 12.6%–41.4%) and the PNV was 99.6% (95% CI: 97.8 -100). The LR + was 8.58 (95% CI: 7.1 -10.4), increasing further with higher thresholds. In contrast, the LR- for this threshold was 0.10 or 1/10 (95% CI: 0.02 – 0.7). According to sensibility, sensitivity, PPV, PNV, LR + and LR-, AQC itself with a threshold of 4.6, might identify LC patients with both high sensitivity (64%) and confirm absence of LC with a high specificity (89.4%). Additionally, AQC itself might identify PNL with a sensitivity of 90.91% and also confirm subjects without PNL, with a high specificity of 89.40%, with a threshold of 4.6.

### Combining (Multiple) testing

In order to improve the diagnostic accuracy, a combining multiple test was applied as two-test parallel combination and two-test series combination.

Tests performed in parallel for LC, using DR70 and AQC showed a sensitivity of 92.0% (95% CI: 79.4 -100.0) and specificity of 80.9% (95% CI: 76.2 -85.7) (Table 3A). Combining testing in parallel increased the sensitivity of AQC for LC, since 64.0% to 92.0%, while the PPV showed a decreasing, since 34.8% to 29.9%. The NPV showed not significant changes. For DR70, the parallel testing showed a non significant decreasing of sensitivity since 95.83% to 92.0% and specificity, since 91.9% to 80.9%.

**Table 3:** **Combining multiple test as two-test parallel (A) and serial (B) combination**

**Test A**	**Sensitivity % (95% CI)**		**Specificity % (95% CI)**		**PPV % (95% CI)**		**PNV % (95% CI)**	
	**LC**	**PNL**	**LC**	**PNL**	**LC**	**PNL**	**LC**	**PNL**
**DR70**	95.8 (78.9- 99.9)	27.3 (6.0-61.0)	91.9 (88.1-94.8)	91.9 (88.1-94.3)	50.0 (35.1 -65.1)	11.5 (2.4 -30.2)	99.6 (97.8 -100)	97.0 (94.2 -98.7)
**AQC**	64.0 (42.5- 82.0)	90.9 (58.7-99.8)	89.4 (85.2-92.7)	89.4 (85.2-92.7)	34.8 (21.2 -50.4)	25.0 (12.6 -41.4)	96.6 (93.6 -98.6)	99.6 (97.8 -100)
**Parallel combined test**	92.0 (74.4 -100)	91.2 (83.0-99.5)	80.92 (76.2-85.7)	80.92 (76.2-85.7)	29.9 (19.0 -40.7)	49.1 (39.1 -59.1)	99.1 (97.7 -100)	97.9 (95.8 -99.9)
**Test B**	**Sensitivity % (95% CI)**		**Specificity % (95% CI)**		**PPV % (95% CI)**		**PNV % (95% CI)**	
	**LC**	**PNL**	**LC**	**PNL**	**LC**	**PNL**	**LC**	**PNL**
**DR70**	95.8 (78.9- 99.9)	27.3 (6.0-61.0)	91.9 (88.1-94.8)	91.9 (88.1-94.3)	50.0 (35.1 -65.1)	11.5 (2.4 -30.2)	99.6 (97.8 -100)	97.0 (94.2 -98.7)
**AQC**	64.0 (42.5- 82.0)	90.9 (58.7-99.8)	89.4 (85.2-92.7)	89.4 (85.2-92.7)	34.8 (21.2 -50.4)	25.0 (12.6 -41.4)	96.6 (93.6 -98.6)	99.6 (97.8 -100)
**Serial combined test**	60.0 (38.8 -81.2)	35.1 (21.8 -48.4)	97.2 (95.1 -99.3)	97.2 (95.1-99.3)	65.2 (43.6 -86.9)	71.4 (52.9 -90.0)	96.5 (94.2 -98.8)	88.1 (84.4 -91.9)

LC: Lung Cancer; PNL: Pre Neoplastic Lesion; PPV: Positive Predictive Value; PNV: Negative Predictive Value; CI: Confidence Interval.

Additionally, the PPV decreased significantly, since 50.0% to 29.9%. However, the PNV for DR70 showed not significant changes.

On the other hand, tests performed in parallel for PNL, using DR70 and AQC showed an increasing of sensitivity in both assay (DR70 and AQC). For DR70 the sensitivity increased since 27.3% to 91.23% (95% CI: 83.0 -99.5), while the specificity showed non statically decreasing; since 91.9% to 80.92% (95% CI: 76.2 -85.7). AQC didn’t show significant differences with the parallel test both sensitivity and specificity. However, both test showed a significant increasing in the PPV, since 11.5% for DR70 and 25.0% for AQC to 49.1% for parallel testing.

Additionally, an illustration of the effects of serial combination testing is shown in Table 3B for a screening protocol with DR70 and AQC. The serial testing for LC, results in decreasing sensitivity, for both test, especially for DR70, but with and increasing of the PPV to 65.2% since 50.0% for DR70 and since 34.8% for AQC. The specificity showed a significant increasing, since 91.9% for DR70 and 89.4% for AQC to 97.2% (95% CI: 95.1%–99.3%). PNV didn’t show significant changes with serial combination. On the other hand, the serial combination testing for PNL, showed a significant decreasing of sensitivity for AQC, since 90.9% to 35.1%, and a non significant increasing for DR70. However, the specificity showed a significant increasing to 97.2% (95% CI: 95.1%–99.3%), since 91.9% for DR70 and since 89.4% for AQC. Additionally, PPV showed a significant increasing since 11.5% for DR70 and 25.0% for AQC to 71.4.

## Discussion

The aim of this study was to explore two biomarkers as potential tools to manage of lung cancer (LC) risk. Our results showed that AFB could identify PNL (metaplasia and dysplasia) better than WLB by its self, in patients with high likelihood to malignance according AQC and DR70. The AFB was able to detect 12.25% of PNL (12 out of 98) in a healthy population, with LC risk, according to the LC risk survey. Additionally, AQC might identify PNL with a high sensitivity of 90.91%, specificity of 89.4% and a PPV of 25.0% and a PNV of 99.6%. According to these results, this test might be better confirming subject without PNL, with a high specificity and PNV. For PNL, AQC by itself resulted in a better sensitivity as compared to DR70 (27.3%). However, both of them by itself showed low PPV (11.5% for DR70 and 25% for AQC). Due to the low overall sensitivity of DR70 for PNL, the assay should be used with AQC to improve the sensitivity, especially as a parallel combination protocol. A parallel combination would be able to confirm positivity for PNL, with a sensitivity of 91.2% and a PPV of 49.1%.

For LC, according to sensitivity, PPV, LR + and LR-, our results showed that DR70 might contribute to the confirmation of LC diagnosis and identification of the patients with advanced LC, with a sensitivity of 95.83% and a PPV of 50%. However, the test would be better in negativity LC cases with specificity of 91.87% and PNV of 99.6%. Therefore, DR70 might be used mainly as a complementary tool to confirm LC diagnosis and identify patients with advanced LC. On the other hand, AQC resulted in a high sensitivity (64%) and specificity (89.4%) for LC with a higher PNV (96.6%) rather than PPV (34.8%). This finding confirmed that AQC would support in the detection and confirmation of positive LC cases and especially discard the presence of malignance, with high precision (specificity of 89.4% and PNV of 96.6%).

For LC and PNL, both test (AQC and DR70) might improve the diagnosis of LC, especially as combined protocols. Parallel combination might be used as complementary tools, mainly as parallel test with a sensitivity of 92.0% and 91.2% for LC and PNL, respectively and high specificity.

## Conclusion

In conclusion, as a pre-screening tool for LC, both biomarkers might be employed with a high specificity and sensitivity as complementary tools to detect LC.

The prevalence for LC and PNL in this population was 3.74%. Although the population studied represented a high risk population, the prevalence that was resulted from this study might be related to the initial step of volunteer recruitment. These were consisted of: adequate survey, appropriate selection of biomarkers and the inclusion of ordinary individuals and clinicians in the study in order to detect the right candidates with high risk for LC in both cities, Santiago and Antofagasta.

Finally, it is important to note that although both tests together might be able to detect and confirm LC and or PNL, this proposal is not necessarily a diagnostic improvement but can be used as an additional tool in the detection of LC or preneoplastic lesions. Screening tests might be combined to improve the efficiency of LC or PNL diagnosis.

These results might improve general health standards by improving the early detection of LC, especially in high risk people. In addition, this work provides scientific and clinical information for Chilean health authorities to include LC in the AUGE government programme, which provides additional health services for patients. Chile needs to formulate policies concerning screening for LC and PNL since there are a lot of benefits in it for the patients and its costs to the individuals are reasonable while providing a good quality of life for the patients.

## Methods

The study designed as double blind, enrolled people (364) from the Metropolitan (Santiago city, N = 224) and Antofagasta (N = 140) regions, with medium or high risk for LC, according to a LC Risk Survey [36, 37]. Healthy voluntaries, without symptoms and diagnosis of LC, were male/female, with aged 40 years or older; family history of LC, non-smokers, ex-smokers and ever smokers; exposed naturally to environmental air pollution (Santiago) or to dw-As (Antofagasta) for at least 10 years. In addition, we enrolled subjects who were suspected of having lung cancer (N = 24) based on their clinical symptoms, without diagnosis and treatment at the moment to enter to the study. These patients did not have previous tests performed on them such as cytology or CT. After of a healthy control and interview with a general practitioner, informed consent was signed and a sputum sample was obtained prospectively using inhalation of nebulised 3% hypertonic saline. Subjects were instructed to cough. Additionally, blood sera samples were obtained of each volunteer for determine the DR70 levels. The concentrations of DR70 in the sera were obtained from a standard curve, which results from the extinctions of calibrators provided with the kit.

AQC, was done according to LungSignTM test (Perceptronix Medical Inc.), as described by Kemp et al. [25] and the sputum generation, was induced with inhalation of 3% saline solution. The optimum cut-off level for AQC of 4.6 was determined selecting the point on the ROC (Receiver Operating Characteristic) curve which maximised both sensitivity and 1–specificity, using MedCalc statistical software 12.1 (MedCalc Inc., Mariakerke, Belgium) with 95% confidence intervals. This value was equivalent to the threshold recommended by Perceptronix Medical Inc. (threshold = 4.6).

Furthermore, for DR70, serum was separated and tested along with the calibrators according to AMDL Diagnostics Onko Sure protocol (Radient Pharmaceuticals) as described in Adonis et al. [19] and Hatton et al. [38]. The best cutoff values for the DR70 immunoassay was obtained with the ROC curve analysis using the MedCalc statistical software 12.1 with 95% confidence intervals. The threshold of 1.0 was associated with best sensitivity and 1-specificity and according with the protocol of Onko Sure protocol. The Youden Index was used as measure of the ROC curve, in order to get the effectiveness of diagnostic and select the optimal threshold value (cutoff point) for both markers.

According to AQC and DR70, the voluntaries were classified with their risk score (low, medium and high). Participants with positive DR70 test (threshold >1.0), or an increased likelihood of malignance according to AQC (threshold ≥4.6) were invited to have an AFB, using the Onco-LIFE device (Novadaq Inc., Richmond, Canada), under local anaesthesia. However, some volunteers did not agree to be included in the AFB procedure and were studied only with a Computerized Tomography (CT) and were confirmed for LC or another disease as TBC or pulmonary emphysema. The bronchoscopic procedures were carried out under local anaesthesia with or without sedation. The airways were examined by WLB and then by AFB and the visual findings were classified as normal (class I: non visual abnormality), abnormal (class II: airway inflammation, trauma or anatomical abnormalities), suspicious of malignant change (class III: areas suggesting moderate dysplasia or severe dysplasia) and suspicious of invasive tumour or visible tumour (class IV), as described by Lam et al. [31]. Endobronchial mucosal biopsies were taken from all areas that were suspicious under WLB or AFB. In addition, surveillance biopsies were taken from epithelium with normal appearance in all subjects. An average of 2–3 biopsies was taken from each of the participants. In addition to CT, the pathology assay, in the biopsies collected during the bronchoscopic procedure, confirmed the final diagnosis as normal, pre neoplastic lesion (PNL) (metaplasia or dysplasia) or LC. No adverse events were reported during the study.

Categorical variables were analysed by Fisher’s exact test. The sensitivity and specificity were assessed for each test and was used in analysis as a single test; two-test parallel combination and two-test series combination [39, 40]. Receiver operating characteristic (ROC) curve analysis was carried out using the MedCalc statistical software 12.1 with 95% confidence intervals.

A logistic regression was used combining both biomarkers. The diagnostic cut-off value was determined from ROC curve and the value was compared with results obtained from parallel testing and serial testing. Sensitivity, specificity, predictive values (both positive and negative) and the Youden’s index were calculated for AQC and DR70 using Epidat 3.1.
